# A CRISPR-Cas12b–Based Platform for Ultrasensitive, Rapid, and Highly Specific Detection of Hepatitis B Virus Genotypes B and C in Clinical Application

**DOI:** 10.3389/fbioe.2021.743322

**Published:** 2021-10-07

**Authors:** Xu Chen, Yan Tan, Shuoshi Wang, Xueli Wu, Rui Liu, Xinggui Yang, Yi Wang, Jun Tai, Shijun Li

**Affiliations:** ^1^ Central Laboratory of the Second Affiliated Hospital, Guizhou University of Traditional Chinese Medicine, Guiyang, China; ^2^ Guizhou Provincial Center for Clinical Laboratory, Guiyang, China; ^3^ Public Health School, Guizhou Medical University, Guiyang, China; ^4^ Experimental Research Center, Capital Institute of Pediatrics, Beijing, China; ^5^ Department of Otolaryngology, Head and Neck Surgery, Children’s Hospital Capital Institute of Pediatrics, Beijing, China; ^6^ Guizhou Provincial Centre for Disease Control and Prevention, Guiyang, China

**Keywords:** hepatitis B virus, CRISPR, Cas12b, multiple cross displacement amplification, lateral flow biosensor

## Abstract

Hepatitis B virus (HBV) is one of the most dangerous and prevalent agents that causes acute and chronic liver diseases in humans. Genotyping plays an important role in determining clinical outcomes and response to antiviral treatment in HBV–infected patients. Here, we first devised a CRISPR–based testing platform, termed “CRISPR-HBV,” for ultrasensitive, highly specific, and rapid detection of two major HBV genotypes (HBV-B and HBV-C) in clinical application. The CRISPR-HBV employed multiple cross displacement amplification (MCDA) for rapid preamplification and then Cas12b–based detection for decoding the targets. Finally, the detection result was read out with real-time fluorescence and a lateral flow biosensor. The sensitivity of CRISPR-HBV was 10 copies per test. The specificity was one hundred percent, and no cross reactions were observed in other HBV genotypes and pathogens. The whole detection process, including DNA template extraction (15 min), preamplification reaction of MCDA (30 min at 65°C), CRISPR-Cas12b–based detection (5 min at 37°C), and results readout (∼2 min), could be completed within 1 h. The feasibility of the CRISPR-HBV assay for genotyping HBV-B and -C as successfully validated with clinical samples. Hence, the CRISPR-HBV assay has remarkable potential to develop a point-of-care testing for identifying and distinguishing HBV genotypes B and C in clinical settings, especially in resource-scarcity countries.

## Introduction

Hepatitis B virus (HBV) is one of the main pathogens that can cause severe liver diseases, such as liver failure, liver cirrhosis, and hepatocellular carcinoma, which could be transmitted through exposure to infected blood and body fluids (Nelson et al., 2016; Nguyen et al., 2020; Bertoletti and Bert, 2018). Approximately 257 million people are living with chronic HBV infection, and it causes 700,000 deaths annually worldwide according to the World Health Organization (WHO) reports (World Health organization, 2017). Therefore, it is still a major public health concern in the world. It has been reported that there are at least eight genotypes (A–H) that are divergent by >8% across the entire genome (Zhao et al., 2010). In clinical practice, the genotype characteristic is essential for finding the severity of HBV infection and response to antiviral therapy in hepatitis B patients (Wang et al., 2015; Zhao et al., 2010). China has a high incidence of HBV, and genotypes B and C were identified as the most common agents (more than 95%) (Lin and Kao, 2011; Wang et al., 2019; Su et al., 2020). HBV genotype C causes more severe liver fibrosis, which more easily progresses to hepatocellular carcinoma, than genotype B infection (Zhao et al., 2010). Moreover, genotype C is also related to a less response to antiviral treatment than genotype B (Zeng et al., 2008). Hence, identification and discrimination of HBV genotypes B and C is essential for the follow-up clinical therapy and management of HBV–infected patients in the Asia–Pacific region.

Real-time polymerase chain reaction (PCR), restriction fragment length polymorphism (RFLP) analysis, and DNA direct sequencing have been widely used for genotyping in clinical practice (Irshad et al., 2016; Inoue and Tanaka, 2020; Wang et al., 2015). However, these diagnostic services require expensive apparatus, skilled personnel, and specialized labs, which may not be available in many resource-limited countries. Besides, it is also time-consuming. Hence, devising a rapid, specific, sensitive, and easy-to-use assay for genotyping HBV is essential for the follow-up therapy in HBV–infected patients.

A CRISPR/Cas (Clustered Regularly Interspaced Short Palindromic Repeat and CRISPR-Associated Protein) system was discovered first in the adaptive immunity of archaea and bacteria for eliminating invading nucleic acids (Yao et al., 2018). The Cas effector proteins navigated with guide RNA (gRNA) to target and cleave an invading nucleic acid. Over the last few years, CRISPR/Cas systems, such as CRISPR/Cas9, CRISPR/Cas13, and CRISPR/Cas12, have become prominent tools for genome editing. Recently, the CRISPR/Cas system has displayed potential for the development of next-generation nucleic acid–diagnostics methodology owing to its high sensitivity, specificity, and reliability (Gootenberg et al., 2017). The principle of detection with a CRISPR/Cas platform is based on the *trans*-cleavage activities of Cas nucleases, such as Cas13, Cas12a, and Cas12b, which have the ability to nonspecifically and indiscriminately cleave surrounding nontarget ssRNA and ssDNA when Cas nucleases bound to the target sequence under the guidance of CRISPR RNA (gRNA) (Gootenberg et al., 2018). Combined with isothermal amplification, Cas13, Cas12a, and Cas12b have been used to devise rapid target nucleic acid–detection platforms, such as SHERLOCK (specific high-sensitivity enzymatic reporter unlocking), DETECTR (DNA endonuclease-targeted CRISPR *trans*-reporter), and HOLMESv2 (one-hour low-cost multipurpose highly efficient system v2) (Chen et al., 2018; Kellner et al., 2019; Li et al., 2019), respectively. These assays can accurately, sensitively, and rapidly detect various targets, including RNA and DNA viruses, bacteria, DNA genotypes, drug-resistant genes, and cancer mutations (Liang et al., 2019).

**FIGURE 1:**
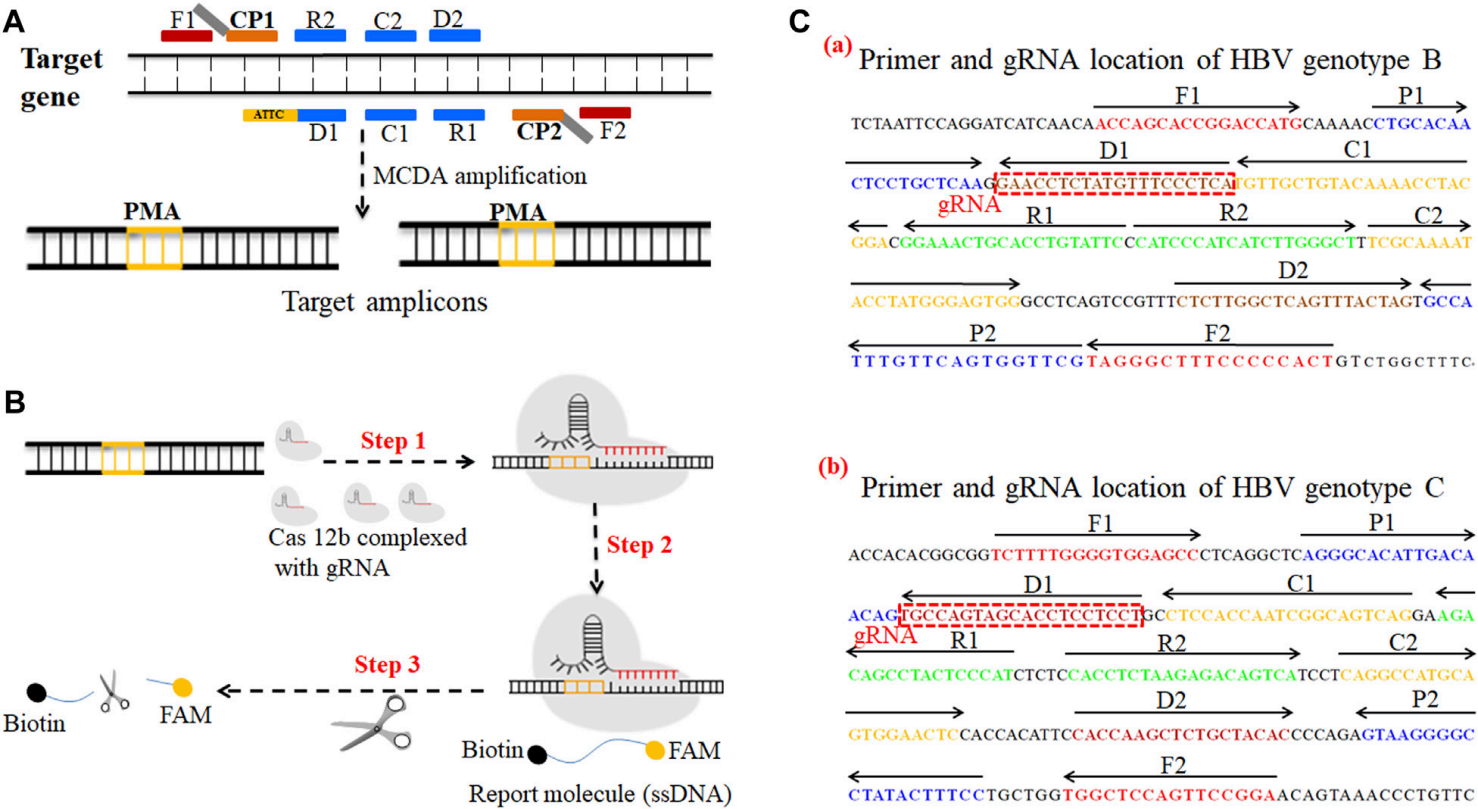
A schematic illustration of the principle of the CRISPR-HBV system. **(A)** Schematic illustration of the principle of MCDA with the modified primer. The amplification primer D1 was modified with a PAM site (TTC). After amplification, a CRISPR-Cas12b recognition site was constructed in the target amplicons. **(B)** Schematic illustration of the CRISPR-Cas12b detection system. Upon recognition of the matching target sequence, the CRISPR-Cas12b complex cleaves a single-stranded DNA reporter molecule. © Sequences and location of the *S* gene of HBV genotypes B and C used to devise the MCDA primers and gRNAs. The sites of MCDA primers are underlined, and the gRNAs are in boxes. Right arrows and left arrows indicate the sense and complementary sequences which were used in this study, respectively.

**FIGURE 2:**
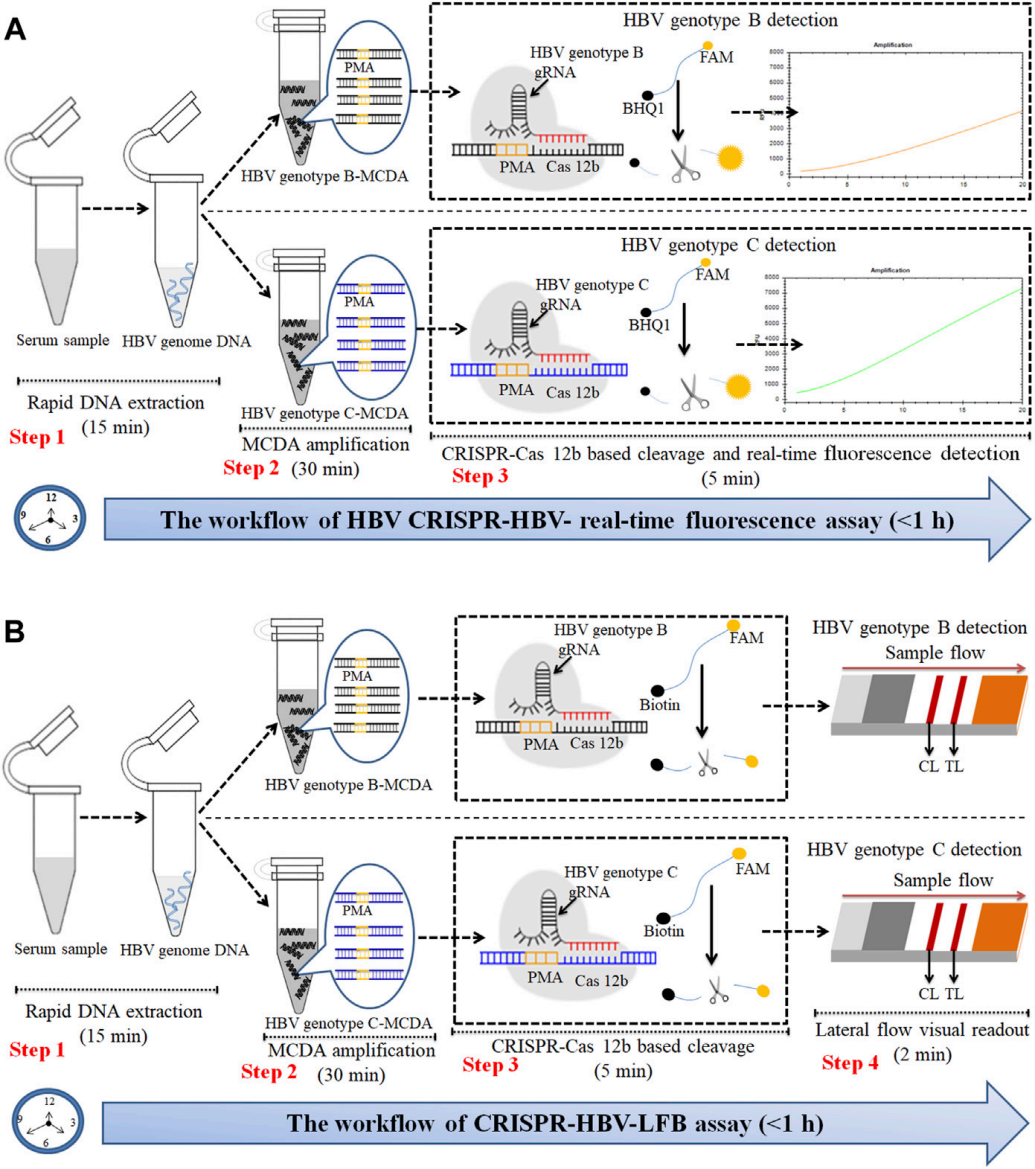
An outline of the CRISPR-HBV workflow. **(A)** The CRISPR-HBV RTF assay employs three closely linked steps: DNA extraction (step 1), MCDA (step 2), and CRISPR-Cas12b cleavage and RTF readout (step 3). The whole detection process could be completed within 1 h. **(B)** CRISPR-HBV–LFB assay employs four closely linked steps: DNA extraction (step 1), MCDA (step 2), CRISPR-Cas12b cleavage (step 3), and LFB readout (step 4). The whole detection process could be completed within 60 min.

Currently, most of the CRISPR/Cas detection platforms rely on expensive fluorescence-based instruments (Liang et al., 2019), which can cause practical inconvenience and make the platform less robust in resource-limited settings. To overcome previous limitations, the nanoparticle-based lateral flow biosensor (LFB) was successfully devised and used to identify CRISPR/Cas detection results due to its visual readout, low-cost, stability, simplicity, and easy-to-use characteristics (Mukama et al., 2020). To increase the sensitivity of CRISPR/Cas diagnostics, the target gene will be preamplified by PCR or isothermal amplification (Pickar-Oliver and Gersbach, 2019). Multiple cross displacement amplification (MCDA), an innovative nucleic acid isothermal amplification technique, has been applied as an attractive alternative to the traditional PCR–related technique and has potential to develop a point-of-care (POC) testing owing to its rapidity, simplicity, and easy operation (Wang et al., 2017; Li et al., 2020). In the current study, we integrated the preamplification step of MCDA with CRISPR-Cas12b–LFB readout to develop a novel assay termed “CRISPR-HBV” for ultrasensitive, highly specific, and rapid detection of HBV genotypes B and C. In addition, a protospacer adjacent motif (PAM) site (TTC) for the CRISPR-Cas12b–based assay was added into the MCDA primers for detecting any sequences that meet the demand of the primer design (even if the target sequences do not contain any PAM sites). We illustrated the principle of the CRISPR-HBV assay in Figures 1 and 2 and validated its feasibility in genotyping of HBV genotypes B and C with clinical specimens.

## Materials and Methods

### Reagents and Instruments

The universal isothermal amplification kits, colorimetric indicator (malachite green), gold nanoparticle–based LFB, and CRISPR-Cas12b protein (C2c1) were obtained from HuiDeXin Biotechnology (Tianjin, China). Anti-FAM (rabbit anti-fluorescein antibody) and biotin-BSA (biotinylated bovine serum albumin) were obtained from Abcam Co., Ltd. (Shanghai, China). The LFB materials, including a sample pad, an absorbent pad, a conjugate pad, a nitrocellulose membrane (NC), and a backing card, were purchased from the Jie-Yi Biotechnology. Co., Ltd. (Shanghai, China). A dye (crimson red) and streptavidin-coated gold nanoparticles (size, 34.46 ± 4.34 nm; extinction coefficient, 6.0 × 10^9^ M^-1^ cm^-1^ at 506 nm) were purchased from Bangs Laboratories, Inc. (Indiana, United States). A real-time turbidimeter (LA-500) was purchased from Eiken Chemical Co., Ltd. (Japan).

### Preparation of Target DNA and Clinical Samples

In this study, the full-length DNA sequences of the *S* gene of HBV genotypes B and C (accession numbers AF100309 and AB014381, respectively) (Zhao et al., 2010) were synthesized and cloned in a pUC57 vector. The two plasmids (genotype B plasmid and genotype C plasmid) were constructed commercially by General Biol (Anhui, China), according to the manufacturer’s instruction. The initial concentration of HBV genotype B and C plasmids was 1 × 10^8^ copies per microliter. The two constructed plasmids acted as the positive control. In addition, the full-length DNA sequences of the *S* gene for HBV genotypes A, D, E, F, G, and H (accession numbers AF090842, X65259, AB032431, AB036910, AF160501, and AY090454) were synthesized and cloned in a pUC57 vector. The 114 suspected HBV–infected serum samples were collected from the Second Affiliated Hospital of Guizhou University of Traditional Chinese Medicine (Guiyang, China) during April 2020 to December 2020. DNA sequencing was used as the gold standard for determining the HBV genotypes. In brief, a portion of the *S* gene was amplified with the primers F 5′-TCT​AGA​CTC​GTG​GTG​GA-3′ and R 5′-GAT​GAT​GGG​ATG​GGA​ATA​CA-3’ (Zhao et al., 2011), and then, the PCR products were sequenced by Dian Medical Laboratory Center Co., Ltd. (Hangzhou, China) and finally analyzed using NCBI genotyping tools (https://www.ncbi.nlm.nih.gov/projects/genotyping/formpage.cgi). Other various pathogens used in the current study are shown in Supplementary Table S2.

### Multiple Cross Displacement Amplification Primers and gRNA Design

The HBV genotype B– and C–MCDA primers were designed using PRIMER PREMIER 5.0 software in accordance with the principle of MCDA reaction based on the genotype B *S* gene (GenBank no. AF100309; 157–837) and the genotype C *S* gene (GenBank no. AB014381; 2,848–3,215, 1–835), respectively. The specificity of each MCDA primer was confirmed with the BLAST analysis tool. In addition, two gRNAs for HBV genotypes B and C based on the *S* gene were designed according to the CRISPR-Cas12b detection mechanism. The locations of each MCDA primer and gRNA are shown in Figure 1C. Moreover, we added the PAM site (TTC) in each MCDA primer for the CRISPR/12b–based assay; the principle of MCDA and the CRISPR-Cas12b–based assay are shown in Figures 1A,B. The MCDA primers and gRNA sequences are shown in Supplementary Table S1. All of the oligonucleotides were synthesized and purified by Genscript Biotech Co., Ltd. (Nanjing, China) with HPLC purification grade.

### Multiple Cross Displacement Amplification

The preamplification step of MCDA was performed with an isothermal amplification kit, according to the manufacturer’s instructions (HuiDeXing Biotech. Co., Ltd. Tianjing, China). In brief, the MCDA reaction system comprise 12.5 μl of 2 × reaction buffer, 0.4 μM each of F1 and F2, 1.6 μM each of CP1 and CP2, 0.8 μM each of C1, C2, D1, D2, R1, and R2, 12.5 μl of 2 × reaction buffer, 1 μl of *Bst* 2.0 DNA polymerase (8 U), 1 μl of AMV reverse transcriptase (10 U) (only used for the RNA template), and a nucleic acid template (1 μl of the standard plasmid and 5 μl of the clinical samples). Finally, 25 μl of double-distilled water was added. The reaction process was carried out with a heat blocker. The amplification results were monitored with real-time turbidity (LA-500) for optimizing the amplification temperature.

### CRISPR-Cas12b–Based Assay

In the current study, Cas12b (C2c1) was used for CRISPR-Cas–based *trans*-cleavage detection. In brief, the CRISPR-Cas12b–gRNA complexes were preassembled as follows: 300 nM CRISPR-Cas12b (C2c1) (Cat no. HT100006) and 100 nM gRNA were preincubated in 1 × HDX buffer at 37°C for 10 min; the complexes should be used immediately or stored at low temperatures (0–4°C) no more than 12 h before use.

The CRISPR-Cas12b–based *trans*-cleavage detection system comprised 2 μl of MCDA products, 1.0 μl of the single-strand DNA reporter molecule (50 μM), 4 μl of the CRISPR-Cas12b–gRNA complex, 25 μl of the 2 × HDX buffer, and distilled water up to 50 μl; then, the detection process was performed at 37°C for 5 min, and the results were analyzed using the real-time fluorescence (RTF) and LFB, respectively. For RTF analysis of the CRISPR-Cas12b *trans*-cleavage detection, the Flu-probe (5′-FAM-TTTTTT-BHQ1-3′, 100 μM) was used. For the LFB assay, the reporter should be replaced with a single-strand DNA reporter molecule (5′-FAM-TTTTTT-Biotin-3′, 50 μM).

### Gold Nanoparticle–Based Lateral Flow Biosensor Design and Assay

**FIGURE 3:**
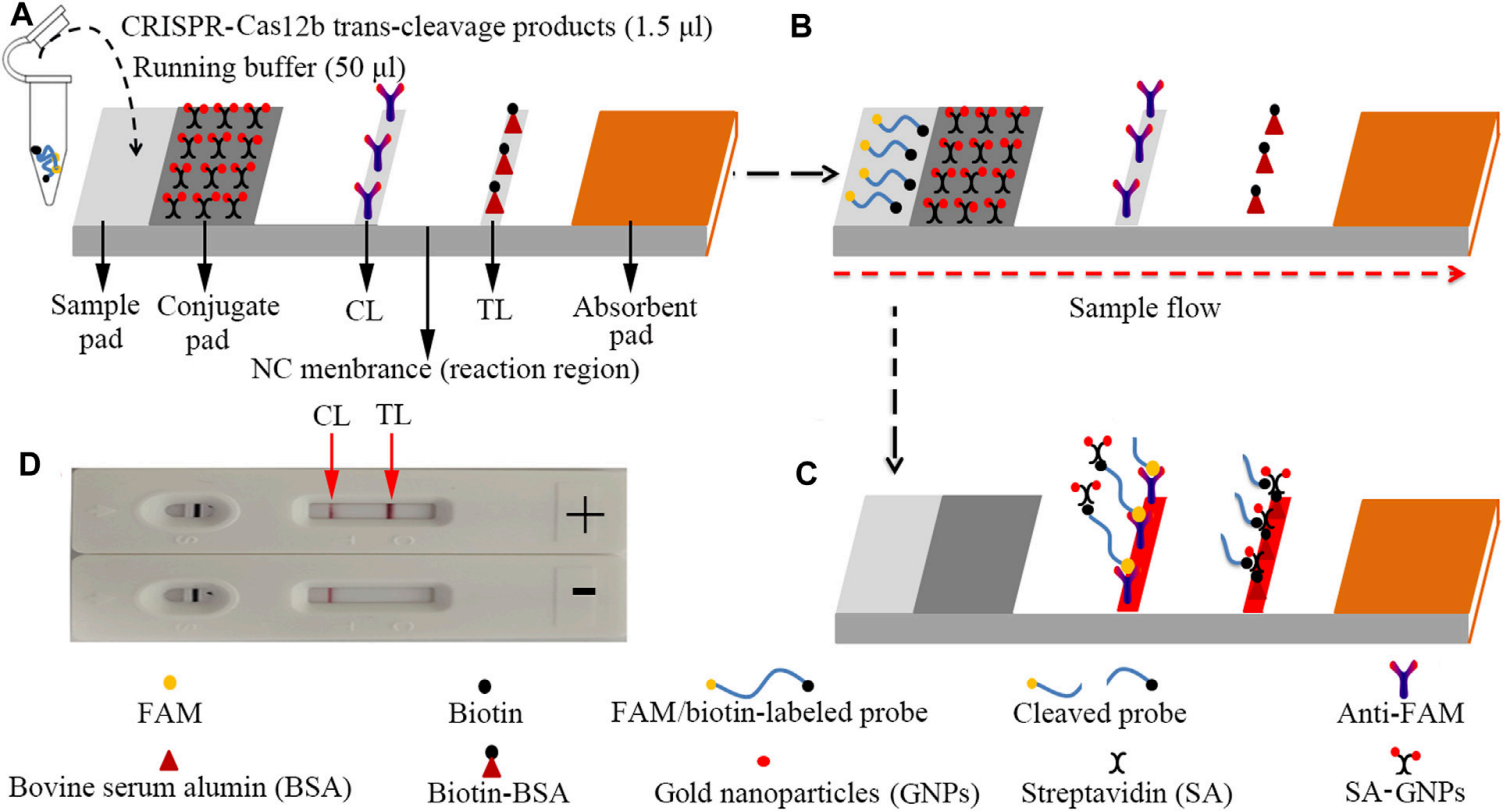
The schematic of the LFB for visualization of HBV genotype B and C products. **(A)** The reaction mixtures (1.5 μl) and the running buffer (50 μl) were deposited on the sample pad. **(B)** The running buffer containing mixtures moved along the LFB owing to capillary action; meanwhile, the dye and streptavidin-coated gold nanoparticles (GNPs) rehydrated in the conjugate region. **(C)** In the positive sample, the ssDNA reporter molecule (5′-FAM-TTTTTT-Biotin-3′) was *trans*-cleaved by the activated CRISPR-Cas12b nuclease and the FAM and biotin were separated. Hence, the biotin–streptavidin–GNPs complex was captured by biotin-BSA at the TL; however, in negative outcomes, the ssDNA reporter molecule was not cleaved and was specifically captured by the anti-FAM at the CL. The biotins of the ssDNA reporter molecule bind streptavidin–GNPs for visualization at the CL. **(D)** Interpretation of the CRISPR-HBV assay results. For positive results, the CL and TL appear on the LFB. When only the CL is observed on the LFB, it indicates negative outcomes. CL: control line; TL: test line.

The LFB (size: 60 mm × 4 mm) used in this study is designed and illustrated in Figure 3. In brief, the biosensor was composed of four sections, including a sample pad, a conjugate pad, a reaction region (nitrocellulose membrane), and an absorbent pad. All of them were assembled on a plastic adhesive backing card. The streptavidin–gold nanoparticles (SA-GNPs) were deposited on the conjugate pad. Anti-FAM and biotin-BSA were fixed on the reaction region for a control line (CL) and a test line (TL), respectively, each line separated by 5 mm. The biosensor used in the current study was manufactured by HuiDeXing Biotech. Co., Ltd. (Tianjing, China) in accordance with our experiment. The LFB can be dry-stored at 4°C for 2 years before use.

For the LFB analysis, 1.5 μl of CRISPR-Cas12b *trans*-cleavage products was added on the sample pad; meanwhile, 50 μl of running buffer (100 mM PBS) was dropped on the sample pad. The running buffer containing CRISPR-Cas12b *trans*-cleavage products was absorbed, and the detection results were read out visually on a NC membrane (red line) within 2 min (Figure 3).

### Sensitivity and Specificity of the CRISPR-HBV Assay

For testing the sensitivity of the CRISPR-HBV assay, two standard plasmids, including HBV genotype B and C plasmids, were 10-fold serially diluted from 1.0 × 10^5^ to 1.0 × 10^-1^ copies. The CRISPR-HBV assay was performed as previously described, and then, the results were detected with the RTF and LFB. Three replicates of each dilution were tested.

The *S* gene of HBV genotypes A–H (synthesized sequences) and other non-HBV pathogens (Supplementary Table S2) were used for verifying the specificity of the CRISPR-HBV assay; distilled water (DW) was applied as the blank control (BC). The CRISPR-HBV assay was performed as previously described and then detected with the RTF and LFB. Each test was confirmed at least three times.

### Verification of the Feasibility of the CRISPR-HBV Assay Using Clinical Samples

For further confirming the feasibility of the CRISPR-HBV assay devised in this study, the optimized CRISPR-HBV assay system was assessed with clinical samples. 114 suspected HBV–infected serum samples were collected from the Second Affiliated Hospital of Guizhou University of Traditional Chinese Medicine (Guiyang, China). The CRISPR-HBV operation was performed as described above. Meanwhile, the clinical samples were tested with direct DNA sequencing (Dian Medical Laboratory Center Co., Ltd. Hangzhou, China). Finally, the outcomes of the CRISPR-HBV assay were compared with those of direct DNA sequencing.

## Results

### Overview of the CRISPR-HBV Detection System

The principle of CRISPR-HBV detection system is illustrated in Figures 1 and 2. In brief, the extracted HBV DNA templates were preamplified by the MCDA method. In this detection system, we modified the MCDA primer D1 at the 5′ end with a PAM site (TTC) (Figure 1A) and the HBV-MCDA amplicons contain a newly acquired Cas12b PAM site for the CRISPR-Cas12b–based assay stage (Figure 1B); the PAM site can be applied for location by the corresponding CRISPR-Cas12b/gRNA system (Figure 1B, Step 1). Then, the CRISPR-Cas12b effector was activated for *trans*-cleavage activity, and the single-strand DNA reporter molecules (5′-FAM-TTTTTT-Biotin-3′) were ultrafast digested (Figure 1B, Step 2 and Step 3). After CRISPR-Cas12b cleavage, the reaction mixtures (1.5 μl) and the running buffer (50 μl) were deposited on the sample region of the LFB (Figure 3A); the running buffer containing reaction mixtures moved along the LFB owing to capillary action, and the streptavidin–GNPs were rehydrated in the conjugate region (Figure 3B). In negative outcomes, the ssDNA reporter molecule was not cleaved and specifically seized by the anti-FAM at the CL. Hence, the biotins of the ssDNA probe (5′-FAM-TTTTTT-Biotin-3′) bind streptavidin–GNPs for visualization readout at the CL (Figure 3C). However, in the positive sample, the ssDNA reporter molecule was *trans*-cleaved by the activated CRISPR-Cas12b nuclease, and the biotin and FAM were separated. Finally, the biotin–streptavidin–GNPs complex was seized by biotin-BSA at the TL (Figure 3C). The interpretation of the CRISPR-HBV assay using the LFB analysis is displayed in Figure 2B and Supplementary Figure S1. The detection results were reported simultaneously using RTF with the Flu-probe (5′-FAM-TTTTTT-BHQ1-3′); the principle is illustrated in Figure 2A.

### Optimal Reaction Conditions for the CRISPR-HBV Assay

Temperature is critical for isothermal amplification. The reaction temperature of the preamplification stage of MCDA was optimized from 60 to 67°C using HBV genotype B and C plasmids (1.0 × 10^3^ copies per reaction), respectively. The results indicated that 65°C was deemed an optimal reaction temperature for the preamplification step of HBV MCDA (Supplementary Figures S2 and S3). Then, the reaction time (1, 2, 5, 10, and 20 min) of CRISPR-Cas12b detection was optimized. The results were read out simultaneously with the LFB and RTF. As shown in Supplementary Figure S4, the stable visual signal was observed by the LFB within 5 min (Supplementary Figures S4A and B) and the fluorescent signal was monitored within 1 min (Supplementary Figures S4C and D).

### Sensitivity and Specificity of the CRISPR-HBV Assay

**FIGURE 4:**
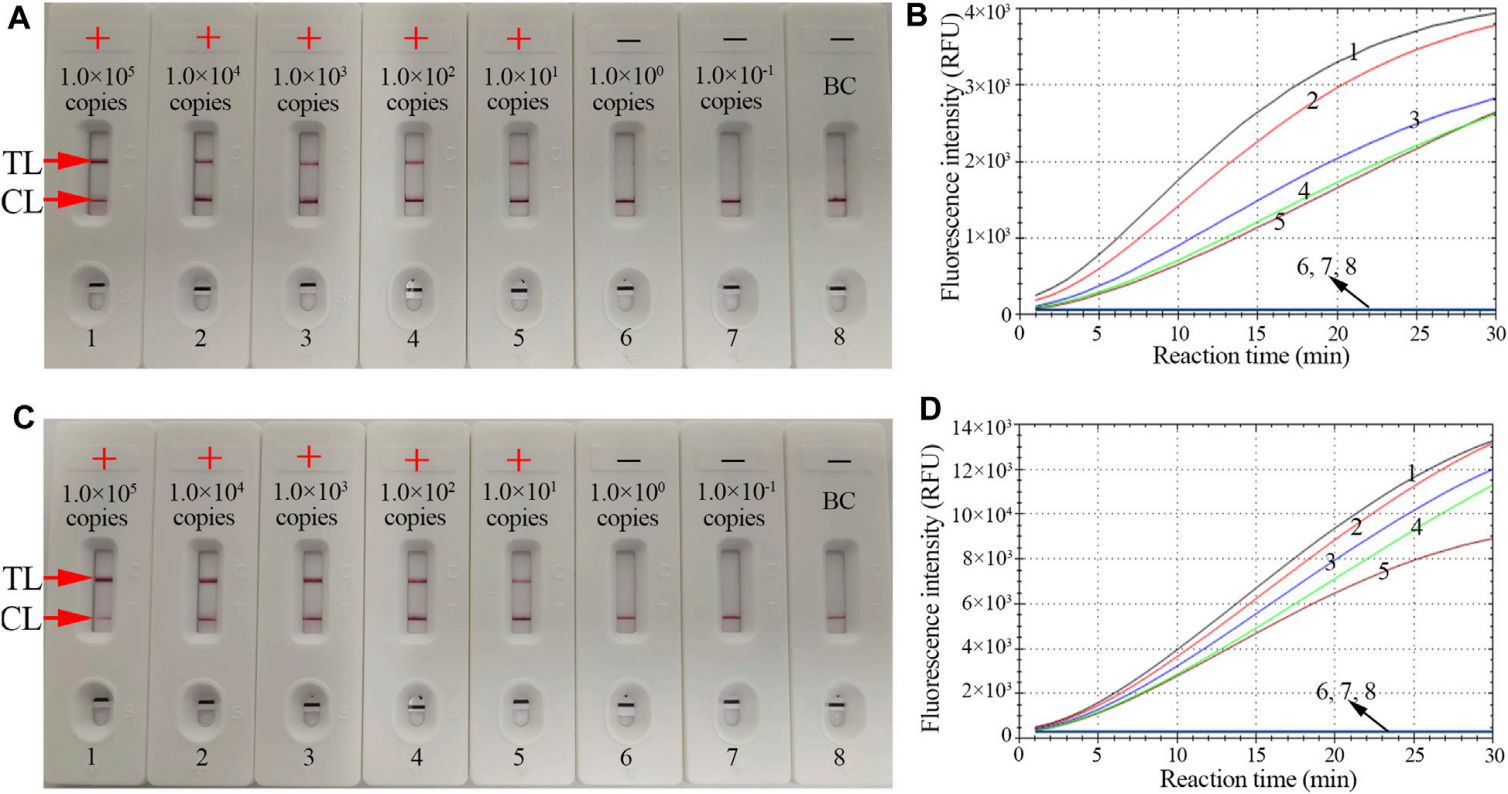
The sensitivity of the CRISPR-HBV assay. LFB and RTF techniques were simultaneously applied for reporting the CRISPR-HBV assay results. LFB **(A)** and RTF **(B)** 1–8 represent the HBV genotype B-*S* plasmid concentrations of 1 × 10^5^, 1 × 10^4^, 1 × 10^3^, 1 × 10^2^, 1 × 10^1^, 1 × 10^0^, and 1 × 10^-1^ copies per reaction and the blank control (DW), respectively. LFB **(B)** and RTF **(D)** 1–8 represent the HBV genotype C-*S* plasmid concentrations of 1 × 10^5^, 1 × 10^4^, 1 × 10^3^, 1 × 10^2^, 1 × 10^1^, 1 × 10^0^, and 1 × 10^-1^ copies per reaction and the blank control (DW), respectively. The LoD of the CRISPR-HBV assay was 10 copies per reaction. CL: control line; TL: test line; “+”: positive; “−”: negative.

The sensitivity of CRISPR-HBV detection was evaluated using HBV genotype B and C plasmids with serial dilutions (ranging from 1.0 × 10^5^ to 1.0 × 10^-1^ copies per reaction). The CRISPR-HBV assay was performed as described above, and the outcomes were read out through the RTF and LFB. For RTF detection, the results indicated that the limit of detection (LoD) of the CRISPR-HBV assay was 10 copies per test (Figures 4B,D), which was completely consistent with the LFB assay (Figures 4A,C).

**FIGURE 5:**
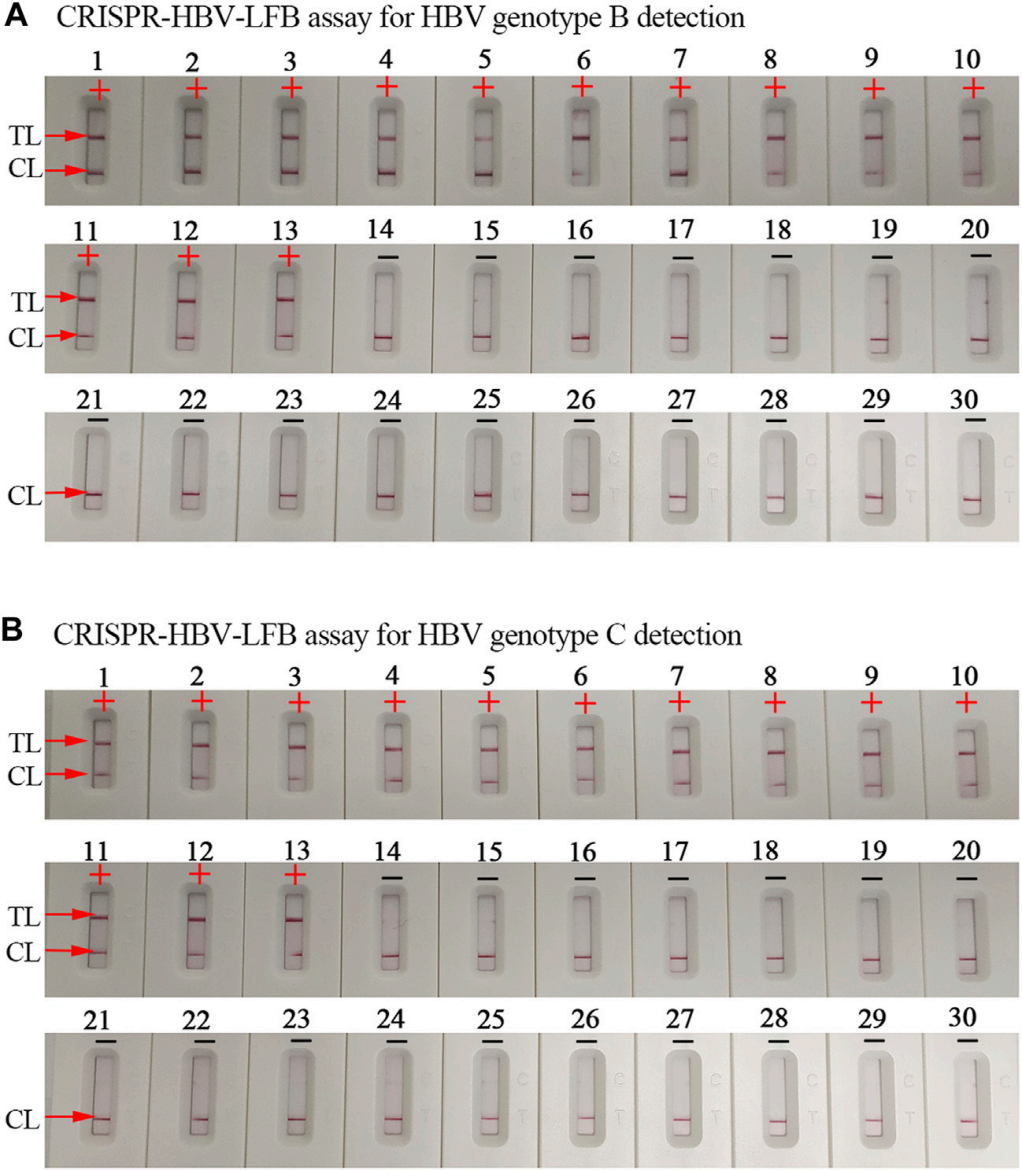
The specificity of the CRISPR-HBV assay. **(A)** The specificity of the CRISPR-HBV–LFB assay for HBV genotype B detection. Biosensor 1, HBV genotype B-*S* plasmid; biosensors 2–9, HBV genotype B agents (clinical samples); biosensors 10–13, HBV genotype B/C agents (clinical samples); biosensor 14, HBV genotype A-*S* plasmid; biosensor 15, HBV genotype C-*S* plasmid; biosensor 16, HBV genotype D-*S* plasmid; biosensor 17, HBV genotype E-*S* plasmid; biosensor 18, HBV genotype F-*S* plasmid; biosensor 19, HBV genotype G-*S* plasmid; biosensor 20, HBV genotype H-*S* plasmid; biosensor 21, hepatitis C virus (standard substance); biosensor 22, human immunodeficiency virus (standard substance); biosensor 23, human rhinovirus; biosensor 24, adenovirus; biosensor 25, *Mycobacterium tuberculosis*; biosensor 26, *Bordetella pertussis*; biosensor 27, *Bacillus cereus*; biosensor 28, *Haemophilus influenzae*; biosensor 29, *Staphylococcus aureus*; and biosensor 30, blank control. CL: control line; TL: test line; “+”: positive; “−”: negative. **(B)** The specificity of the CRISPR-HBV–LFB assay for HBV genotype C detection. Biosensor 1, HBV genotype C-*S* plasmid; biosensors 2–9, HBV genotype C agents (clinical samples); biosensors 10–13, HBV genotype B/C agents (clinical samples); biosensor 14, HBV genotype A-*S* plasmid; biosensor 15, HBV genotype B-*S* plasmid; biosensor 16, HBV genotype D-*S* plasmid; biosensor 17, HBV genotype E-*S* plasmid; biosensor 18, HBV genotype F-*S* plasmid; biosensor 19, HBV genotype G-*S* plasmid; biosensor 20, HBV genotype H-*S* plasmid; biosensor 21, hepatitis C virus (standard substance); biosensor 22, human immunodeficiency virus (standard substance); biosensor 23, human rhinovirus; biosensor 24, adenovirus; biosensor 25, *Mycobacterium tuberculosis*; biosensor 26, *Bordetella pertussis*; biosensor 27, *Bacillus cereus*; biosensor 28, *Haemophilus* influenzae; biosensor 29, *Staphylococcus aureus*; and biosensor 30, blank control. CL: control line; TL: test line; “+”: positive; “−”: negative.

The specificity evaluation of the CRISPR-HBV assay was confirmed using synthesized templates, HBV genotype B– and C–positive clinical samples, and various non-HBV pathogens (Supplementary Table S2). The CRISPR-HBV assay was manipulated by the optimal reaction conditions verified above, and the outcomes were analyzed using the LFB and RTF. The positive outcomes appeared only when the templates were extracted from HBV genotype B or C agents, while other HBV genotypes, non-HBV pathogens, and the blank control presented negative outcomes (Figure 5, Supplementary Figures S5, and S6). No cross reactions were observed in the CRISPR-HBV assay. Therefore, the CRISPR-HBV assay designed in the current study was highly selective to the target sequences.

### Confirming the Feasibility of the CRISPR-HBV Assay in Clinical Samples

To further validate whether the CRISPR-HBV assay could be used for distinguishing between HBV genotypes B and C in clinical application, 114 suspected HBV–infected serum specimens were assayed simultaneously using CRISPR-HBV and direct sequencing. According to the sequencing results, 36 samples were confirmed as genotype B, 28 samples were recognized as genotype C, five samples were identified as genotype B/C, three samples were recognized as genotype D, and 42 samples were tested as non-HBV infection. The results were in accordance with the CRISPR-HBV assay outcomes (Supplementary Table S3). These results indicated that the CRISPR-HBV assay developed in this study could be considered as an advanced technique to distinguish between HBV genotypes B and C in clinical application.

## Discussion

HBV is one of the most dangerous and prevalent agents that causes acute and chronic liver diseases in humans (Pisaturo et al., 2019; Meng et al., 2020). Several investigations have indicated that different HBV genotypes could affect the clinical outcomes and response to antiviral treatment in hepatitis B patients. Genotypes B and C are the two most common agents (accounting for approximately 95%) in China (Lin and Kao, 2011; Wang et al., 2019; Su et al., 2020). Previous studies demonstrated that HBV genotype C was associated with a higher risk of reactivation of hepatitis B, with more severe liver fibrosis, and can more easily progress to hepatocellular carcinoma than genotype B infection (Lin and Kao, 2011). Moreover, HBV genotype C is also associated with a lower response rate to antiviral therapy (Zeng et al., 2008). A previous study demonstrated that genotype B had a better virological response to adefovir dipivoxil therapy than genotype C (Zhao et al., 2010). Hence, detection and discrimination of HBV genotypes B and C are essential for the follow-up clinical treatment and management of HBV–infected patients in the Asia–Pacific region. In the current study, a novel CRISPR-HBV assay, which integrated CRISPR-Cas12b detection with MCDA, was established for identifying and distinguishing HBV genotypes B and C in clinical samples.

An ideal laboratory diagnostic technique for confirmation and distinction of HBV genotypes B and C should be rapid, specific, sensitive, and easy to use. Currently, real-time PCR, RFLP analysis, and direct DNA sequencing have been widely applied for genotyping in clinical practice (Zhao et al., 2011; Wang et al., 2015; Lin and Kao, 2011). However, their use in low- and middle-income regions is significantly limited due to the requirement of expensive instruments and trained experts. A CRISPR/Cas system was discovered first in the adaptive immunity of archaea and bacteria for eliminating invading nucleic acids (Yao et al., 2018; Shiriaeva et al., 2020). The Cas effector proteins were navigated with gRNA to target and cleave an invading nucleic acid (Zhou et al., 2018). Over the last few years, CRISPR/Cas systems, such as CRISPR/Cas9, CRISPR/Cas12, and CRISPR/Cas13, have become a prominent tool for genome editing (Myhrvold et al., 2018; Zhu et al., 2021). Recently, Cas12 and Cas13 effectors were demonstrated to have remarkable potential in developing novel nucleic acid–detection technologies based on their unique characteristic of collateral cleavage of target genes and nonspecific single-stranded nucleic acids (Pickar-Oliver and Gersbach, 2019; Broughton et al., 2020). In this study, we designed successfully the specific gRNAs for HBV genotypes B and C, and the gRNAs navigated Cas12b effector proteins to each of the target sequences. The specificity of the CRISPR-HBV system was strongly confirmed with HBV agents and other pathogens. The results have shown that the CRISPR-HBV assay could clearly distinguish HBV genotypes B and C and have no cross reactions with other pathogens (Supplementary Figures 5S5S6). Hence, the CRISPR-HBV assay displayed a high level of specificity for distinguishing HBV genotypes B and C. Apart from its remarkable specificity, the novel CRISPR-HBV assay could detect as low as 10 copies of genomic DNA per test (Figure 4). Owing to the lack of reference strains for HBV genotypes B and C in our laboratory, the full-length DNA sequences of the *S* gene for HBV genotypes B and C (accession number AF100309 and AB014381, respectively) were synthesized and cloned in the pUC57 vector, respectively. The synthesized plasmids were considered as HBV genotype B and C reference strains. In order to further confirm the sensitivity of the CRISPR-HBV assay in the human serum, the HBV genotype B-*S* or C-*S* plasmids were added into 100 μl serum of a healthy volunteer and the final concentrations of HBV genotype B-*S* or C-*S* genes were made ranging from 1.0 × 10^5^ to 1.0 × 10^-1^ copies/μl, respectively. The nucleic acid was extracted using a Rapid Nucleic Acid Extraction kit (Jiangsu Bioperfectus Technologies Co., Ltd., China) and dissolved into 100 μl distilled water, and then, 1 μl nucleic acid was used for the CRISPR-HBV assay. The results showed that the sensitivity of this assay was also 10 copies (data not shown). More importantly, we also successfully applied the CRISPR-HBV assay to clinical samples. The suspected HBV–infected serum samples were simultaneously detected with the CRISPR-HBV assay and direct DNA sequencing, and the concordance results of the former and latter detections were given (Supplementary Table S3). It is indicated that the CRISPR-HBV assay could be used as a reliable tool for detecting and distinguishing HBV genotypes B and C in clinical application. The CRISPR-HBV assay developed in the current study only has the ability to detect and distinguish HBV genotypes B and C, but not HBV subgenotypes. For conservation across the sequences of entire HBV genotypes B and C, the HBV genotype B and C MCDA primers and gRNA sequences were designed based on the *S* gene that come from the NCBI’s recommended reference sequence strains for genotype B (accession number: AF100309) and genotype C (accession number: AB014381) (Zhao et al., 2010).

In order to improve the sensitivity of the CRISPR-HBV assay, the MCDA method was used for preamplifying the target genes (HBV genotype B-*S* and genotype C-*S*). MCDA was considered as a novel isothermal amplification technique, which is more sensitive than PCR and LAMP assays (Wang et al., 2015; Jiao et al., 2019; Wang et al., 2017). The isothermal amplification of the target sequence through a set of 10 primers spanning 10 different regions of the target gene was conducted (Wang and Wang et al., 2015), which comprised a pair of displacement primers (F1 and F2), a pair of cross primers (CP1 and CP2), and three pairs of amplification primers (C1, C2, D1, D2, R1, and R2) (Figure 1A). More importantly, in the current study, the MCDA-D1 primers were modified with a Cas12b PAM site (TTC); after MCDA, the PAM site was applied for location by the corresponding CRISPR-Cas12b/gRNA complex to target sequences, then the Cas12b nuclease was activated, and the single-strand DNA reporter molecules were ultrafast *trans*-cleaved.

In this report, the gold nanoparticle–based LFB was used for reading out CRISPR-HBV outcomes. Currently, gold nanoparticles have become the most appropriate nanomaterials used as a biosensor owing to high adsorption, the high surface-to-volume ratio, well biocompatibility, and easy synthesis and manipulation (Quesada-González and Merkoçi, 2015; Aldewachi et al., 2017; Huang et al., 2019). The LFB could visually respond to the CRISPR-HBV assay for labeling with biotin-BSA and anti-FAM on the biosensor. In positive results, the ssDNA reporter molecule (5′-FAM-TTTTTT-Biotin-3′) was *trans*-cleaved by the activated CRISPR-Cas12b nuclease, the FAM and biotin probes were separated, and the biotin–streptavidin–GNPs complex was arrested by biotin-BSA and visually observed at the TL. However, in negative results, the ssDNA probe was not cleaved, was specifically captured by the anti-FAM, and was visualized at the CL (Figure 3). Although the RTF technique and the LFB method could be used for identifying the CRISPR-HBV assay, the former requires special instruments and complex operation, whereas the LFB is simple and easy to operate. Hence, LFB was considered as an optimal mean for the CRISPR-HBV assay. The whole detection process, including genomic DNA template preparation (∼15 min), MCDA (30 min), CRISPR-Cas12b/gRNA detection (5 min), and results readout (∼2 min), could be completed within 60 min. Therefore, the CRISPR-HBV assay has potential to develop a POC testing for identifying and distinguishing HBV genotypes B and C in clinical settings, especially in economically impoverished regions of the world. The shortcoming of this study is that we just use CRISPR-Cas12b–based platform for detection of two major HBV genotypes (B and C) in China. Next, we will further utilize the CRISPR-Cas12b–based platform to devise more assays for identifying various kinds of HBV genotypes.

## Conclusion

We combined the preamplification reaction of MCDA with CRISPR-Cas12b and LFB readout to devise a novel assay, termed “CRISPR-HBV,” for ultrasensitive, highly specific, and rapid detection of HBV genotypes B and C in clinical practice. The CRISPR-HBV assay was able to detect 10 copies of genomic DNA per test and has no cross reactions with other agents. The whole assay process could be completed within 60 min and does not require costly facilities. Hence, these traits of our CRISPR-HBV assay have potential to be an important POC testing for identifying and distinguishing HBV genotypes B and C in clinical application, especially in resource-constrained areas.

## Data Availability

The original contributions presented in the study are included in the article/Supplementary Material; further inquiries can be directed to the corresponding authors.
