# Supplementary material for: A CRISPR-Cas12b–Based Platform for Ultrasensitive, Rapid, and Highly Specific Detection of Hepatitis B Virus Genotypes B and C in Clinical Application
Source: Front Bioeng Biotechnol. 2021 Oct 7;9:743322. doi: 10.3389/fbioe.2021.743322 (PMC8529042; doi:10.3389/fbioe.2021.743322)
Supplement: Supplementary file 1 [file Table1.DOCX]

**Supplementary Materials**

**A CRISPR-Cas12b-based platform for ultrasensitive, rapid and highly specific detection of HBV genotype B and C in clinical application**

Xu Chen^1Δ^, Yan Tan^2Δ^, Shuoshi Wang^1^, Xueli Wu^1^, Rui Liu^1^, Xinggui Yang^3^, Yi Wang^4*^, Jun Tai^5*^, and Shijun Li^6*^

^1^ Central Laboratory of the Second Affiliated Hospital, Guizhou University of Traditional Chinese Medicine, Guiyang, Guizhou, 550003, People’s Republic of China

^2^ Guizhou Provincial Center for Clinical Laboratory, Guiyang, Guizhou, 550002, People’s Republic of China

^3^ Public Health School, Guizhou Medical University, Guiyang, Guizhou, 550025, People’s Republic of China

^4^ Experimental Research Center, Capital Institute of Pediatrics, Beijng 100020, P. R. China.

^5^ Department of Otolaryngology, Head and Neck Surgery, Children's Hospital Capital Institute of Pediatrics, Beijing 100020, P. R. China.

^6^ Guizhou Provincial Centre for Disease Control and Prevention, Guiyang, Guizhou, 550004, People’s Republic of China

^Δ^These authors contributed equally.

^*^Corresponding author:

Yi Wang, E-mail: [wildwolf0101@163.com](mailto:wildwolf0101@163.com) (Handing the correspondence)

Jun Tai, E-mail: trenttj@163.com

Shijun Li, E-mail: [zjumedjun@163.com](mailto:zjumedjun@163.com)

**Table S1** The primers and gRNAs used in the current study

| Primers/gRNA | Sequence and modification^a^ | Length^b^ | Target gene and nucleotide numbering |
| --- | --- | --- | --- |
| **HBV genotype B**  F1  F2 | 5ʹ-ACCAGCACCGGACCATG-3ʹ  5ʹ-AGTGGGGGAAAGCCCTA-3ʹ | 17 nt  17 nt | *S* (Genbank Accession No. AF100309) (502-723)  *S* (Genbank Accession No. AB014381) (3066-3215, 1-79) |
| CP1 | 5ʹ-TCCGTAGGTTTTGTACAGCAACA-CTGCACAACTCCTGCTCAA-3ʹ | 42 mer |  |
| CP2 | 5ʹ-TCGCAAAATACCTATGGGAGTGG-CGAACCACTGAACAAATGGC-3ʹ | 43 mer |  |
| C1 | 5ʹ-TCCGTAGGTTTTGTACAGCAACA-3ʹ | 23 nt |  |
| C2 | 5ʹ-TCGCAAAATACCTATGGGAGTGG-3ʹ | 23 nt |  |
| D1 | 5ʹ-**ATTC**-TGAGGGAAACATAGAGGTTC-3ʹ | 20 nt |  |
| D2 | 5ʹ-CTCTTGGCTCAGTTTACTAG-3ʹ | 20 nt |  |
| R1 | 5ʹ-GAATACAGGTGCAGTTTCC-3ʹ | 19 nt |  |
| R2 | 5ʹ-CATCCCATCATCTTGGGCT-3ʹ | 19 nt |  |
| gRNA  **HBV genotype C**  F1  F2  CP1 | 5ʹ-GUCUAGAGGACAGAAUUUUUCAACGGGUGUGCCAAUGGCCACUUUCCAGGUGGCAAAGCCCGUUGAGCUUCUCAAAUCUGAGAAGUGGCACUGAGGGAAACAUAGAGGUUC-3ʹ  5ʹ-TCTTTTGGGGTGGAGCC-3ʹ  5ʹ-TCCGGAACTGGAGCCA-3ʹ  5ʹ-CTGACTGCCGATTGGTGGAG-AGGGCACATTGACAACAG-3ʹ | 111 mer  17 nt  16 nt  38 mer |  |
| CP2  C1  C2  D1  D2  R1  R2  gRNA | 5ʹ-CAGGCCATGCAGTGGAACTC-GGAAAGTATAGGCCCCTTAC-3ʹ  5ʹ-CTGACTGCCGATTGGTGGAG-3ʹ  5ʹ-CAGGCCATGCAGTGGAACTC-3ʹ  5ʹ-**ATTC**-AGGAGGAGGTGCTACTGGCA-3ʹ  5ʹ-CACCAAGCTCTGCTACAC-3ʹ  5ʹ-ATGGGAGTAGGCTGTCT-3ʹ  5ʹ-CACCTCTAAGAGACAGTCA-3ʹ  5ʹ-GUCUAGAGGACAGAAUUUUUCAACGGGUGUGCCAAUGGCCACUUUCCAGGUGGCAAAGCCCGUUGAGCUUCUCAAAUCUGAGAAGUGGCACAGGAGGAGGUGCUACUGGCA-3ʹ | 40 mer  20 nt  20 nt  20 nt  18 nt  17 nt  19 nt  111 mer |  |

^a^, HBV genotype B-D1 and HBV genotype C-D1 primers were modified in linker region with a PAM site (ATTC).

^b^, nt, nucleitide; mer, monomeric unit.

**Table S2** The strains and synthetic templates used in the current study

| NO. | Strains/Templates | Genotype (gene) | Source of strains^a^ | No. of strains | HBV genotypes B and C  DETECTR assay ^b^ | |
| --- | --- | --- | --- | --- | --- | --- |
|  |  |  |  |  | HBV  genotype B | HBV  genotype C |
| 1 | HBV | B (*S* gene) | synthesized | 1 | P | N |
| 2  3  4  5  6  7  8  9  10  11  12  13  14  15  16  17  18  19  20 | HBV  HBV (clinical samples)  HBV (clinical samples)  HBV (clinical samples)  HBV  HBV  HBV  HBV  HBV  HBV  HCV (standard substance)  HIV (standard substance)  Human rhinovirus  Adenoviruses  *Mycobacterium tuberculosis*  *Bordetella pertussis*  *Bacillus cereus*  *Haemophilus influenza*  *Staphylococcus aureus* | C (*S* gene)  B  C  B and C  A (*S* gene)  D (*S* gene)  E (*S* gene)  F (*S* gene)  G (*S* gene)  H (*S* gene)  Unidentified  Unidentified  Unidentified  Unidentified  Unidentified  Unidentified  Unidentified  Unidentified  Unidentified | synthesized  2^nd^ GZUTCM  2^nd^ GZUTCM  2^nd^ GZUTCM  synthesized  synthesized  synthesized  synthesized  synthesized  synthesized  Chinese Academy of Metrology  Chinese Academy of Metrology  GZCCL  GZCCL  GZCDC  GZCDC  GZCDC  ATCC49247  2^nd^ GZUTCM | 1  8  8  4  1  1  1  1  1  1  1  1  1  1  1  1  1  1  1 | N  P  N  P  N  N  N  N  N  N  N  N  N  N  N  N  N  N  N | P  N  P  P  N  N  N  N  N  N  N  N  N  N  N  N  N  N  N |

Notice: ^a^ GZCDC, Guizhou Provincial Center for Disease Control and Prevention; ATCC, American Type Culture Collection; 2^nd^ GZUTCM, the Second Affiliated Hospital, Guizhou University of Traditional Chinese Medicine; GZCCL, Guizhou Provincial Center for Clinical Laboratory.

^b^P, Positive; N, Negative

**Table S3 Detection results of CRISPR-HBV for clinical samples using LFB and real-time fluorescence (RTF) methods**

| Sample NO. | DNA direct sequencing | Results of LFB assay | | Results of RTF assay | |
| --- | --- | --- | --- | --- | --- |
|  |  | Genotype B | Genotype C | Genotype B | Genotype C |
| Test 1-36 | Genotype B | P | N | P | N |
| Test 37-64 | Genotype C | N | P | N | P |
| Test 65-70 | Genotype B/ C | P | P | P | P |
| Test 71 | Genotype D | N | N | N | N |
| Test 72 | Genotype D | N | N | N | N |
| Test 73 | Genotype D | N | N | N | N |
| Test 74-114 | Non HBV infection | N | N | N | N |

**Figure legends**


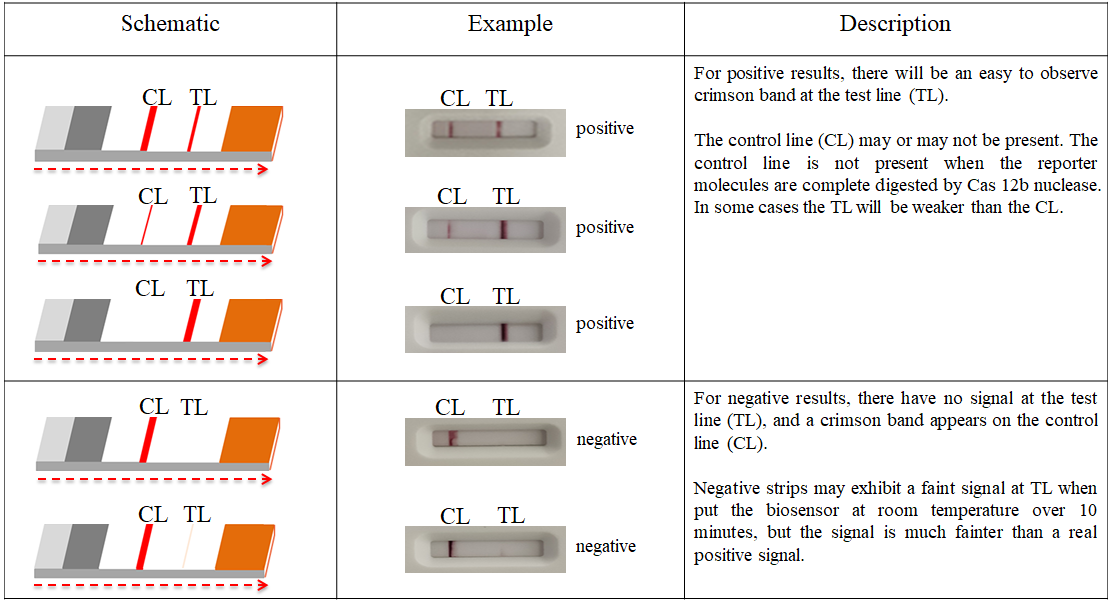


**Fig. S1 Instructions for the interpretation of CRISPR-HBV lateral flow biosensor results.**

**
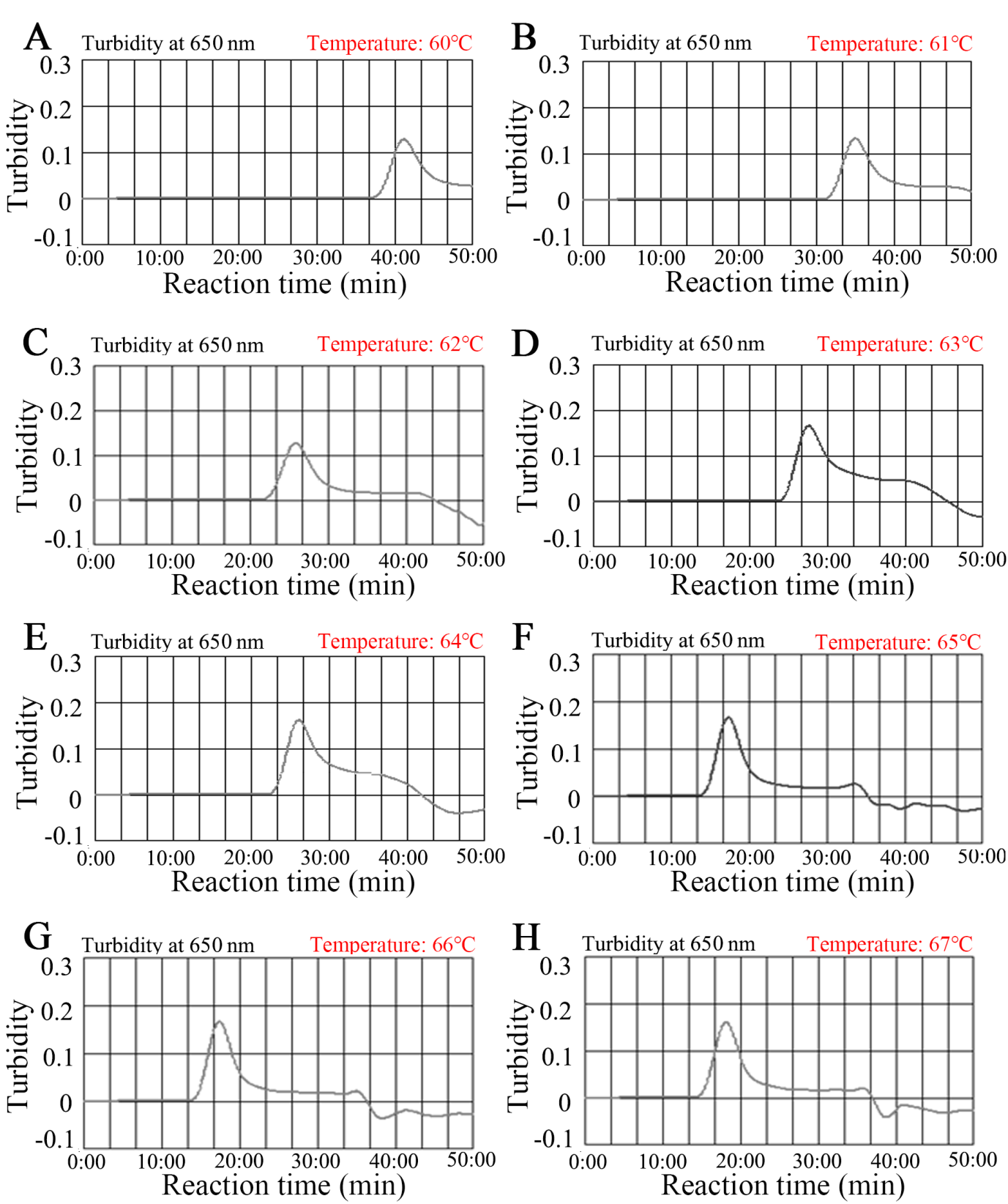
Fig. S2. Optimal amplification temperature for HBV genotype B MCDA primer set.**

The HBV genotype B MCDA reactions were monitored by real-time measurement of turbidity (LA500). Turbidity of >0.1 was considered to be positive, and the threshold value was 0.1. Eight kinetic graphs (**a-h**) were obtained at various reaction temperatures (60-67°C, 1°C intervals) with target DNA template from HBV genotype B-*S* plasmid at the level of 1×10^3^ copies per reaction. The graphs from 65°C to 67°C showed robust amplification.


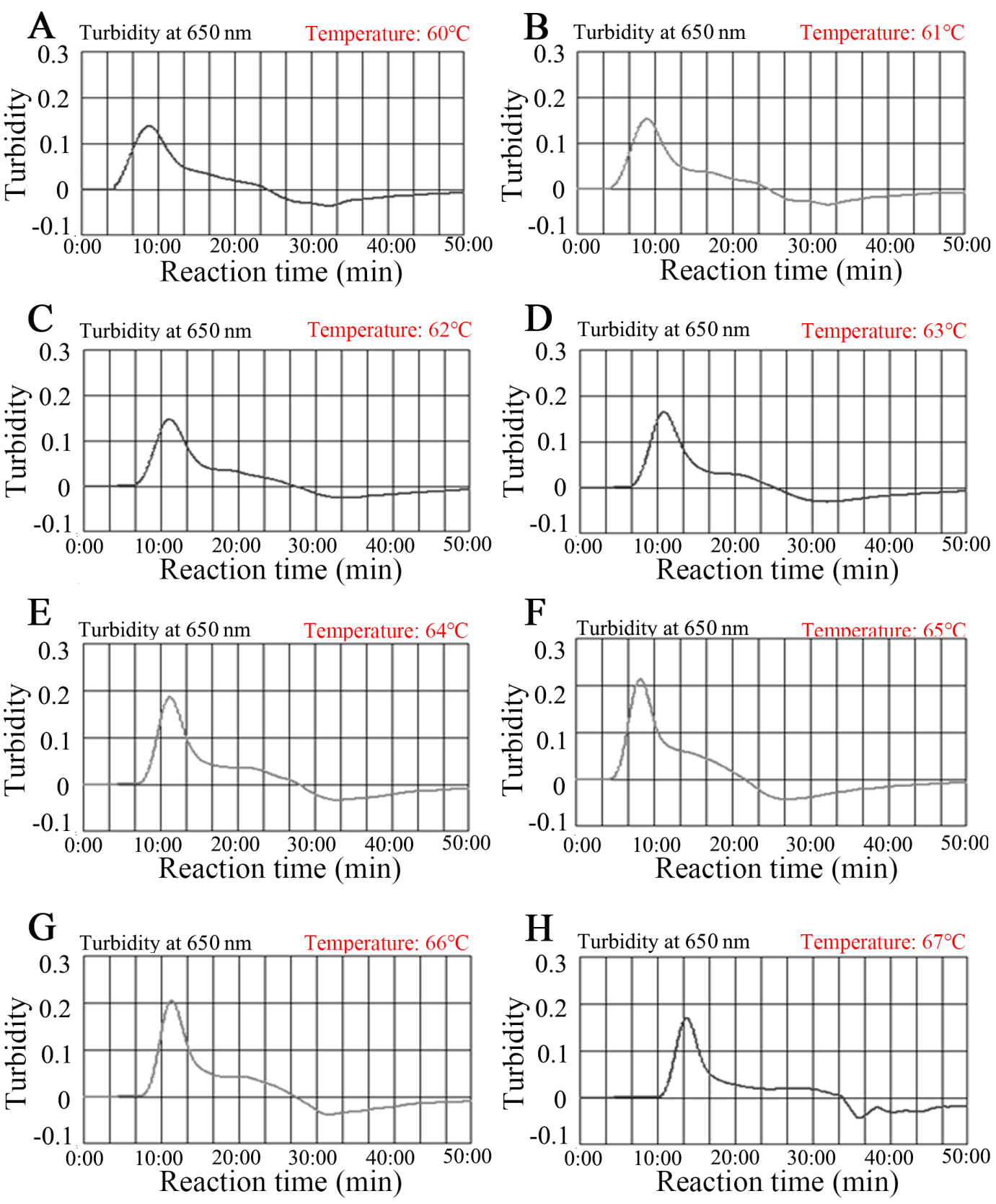


**Fig. S3. Optimal amplification temperature for HBV genotype C MCDA primer set.**

The HBV genotype C MCDA reactions were monitored by real-time measurement of turbidity (LA500). Turbidity of >0.1 was considered to be positive, and the threshold value was 0.1. Eight kinetic graphs (**a-h**) were obtained at various reaction temperatures (60-67°C, 1°C intervals) with target DNA template from HBV genotype C-*S* plasmid at the level of 1×10^3^ copies per reaction. The graphs from 65°C to 66°C showed robust amplification.


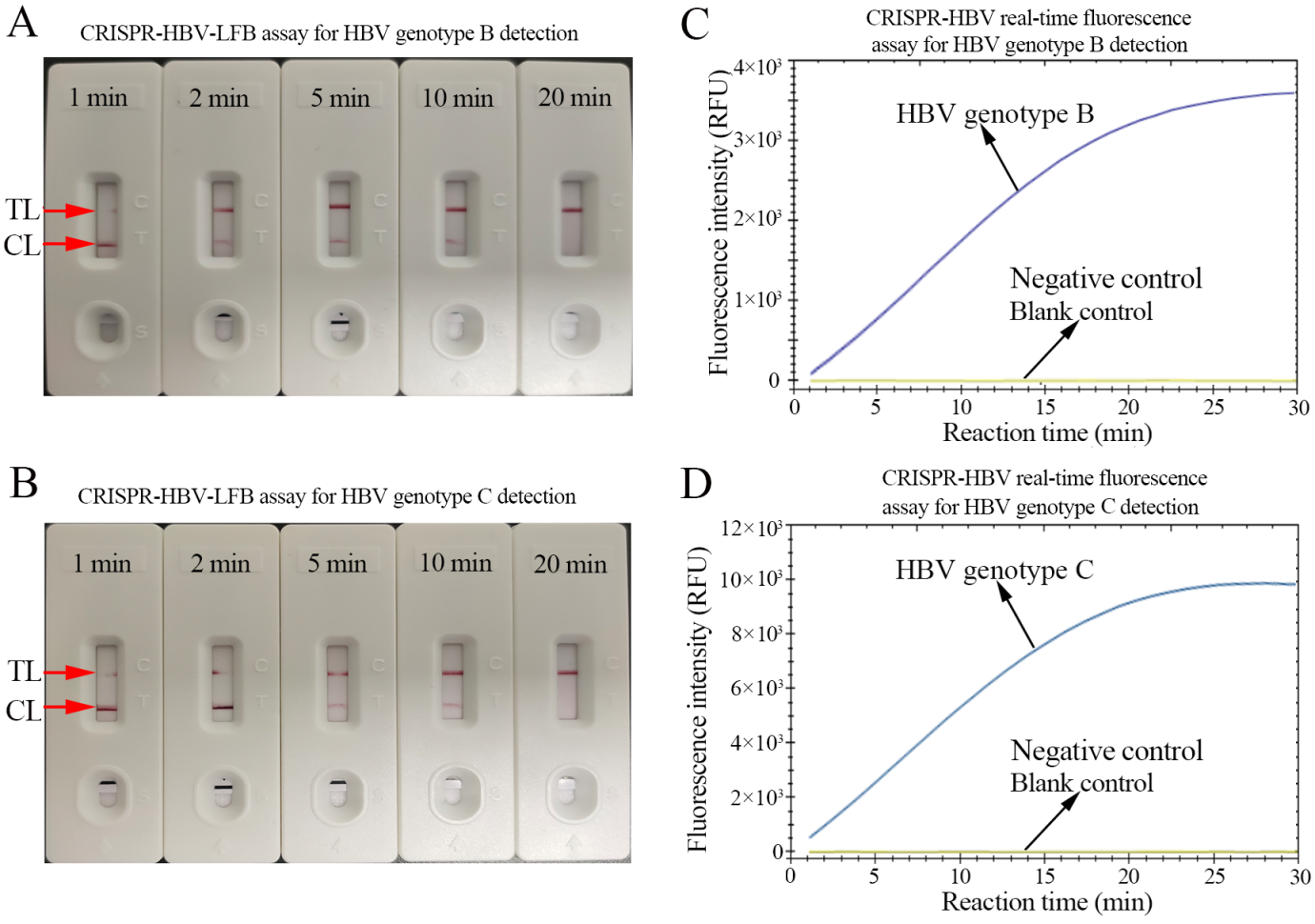


**Fig. S4. Optimal reaction time for CRISPR-Cas12b/gRNA cleavage**

**A** (HBV genotype B detection) and **B** (HBV genotype C detection): Lateral flow biosensor (LFB) was used for reporting the CRISPR-Cas12b/gRNA cleavage results. MCDA products (2 µl) yielded from 1×10^3^ copies of HBV genotype B-*S* plasmid (**A**) and HBV genotype C-*S* plasmid (**B**) were added to corresponding to CRISPR-Cas12b/gRNA reaction. The remarkable signal at the TL and a faint signal at the CL appeared on the biosensor within 5 min, indicating that the ssDNA reporter molecule was sufficiently cleaved. Only the test line was observed on the biosensor when the cleavage reaction lasting 20 min. **C** (HBV genotype B detection) and **D** (HBV genotype C detection): Real-time fluorescence detection was used for reporting the CRISPR-Cas12b/gRNA cleavage results and further confirming the LFB analysis. The fluorescent signal was detected within 1 min, and saturates within 20 min.


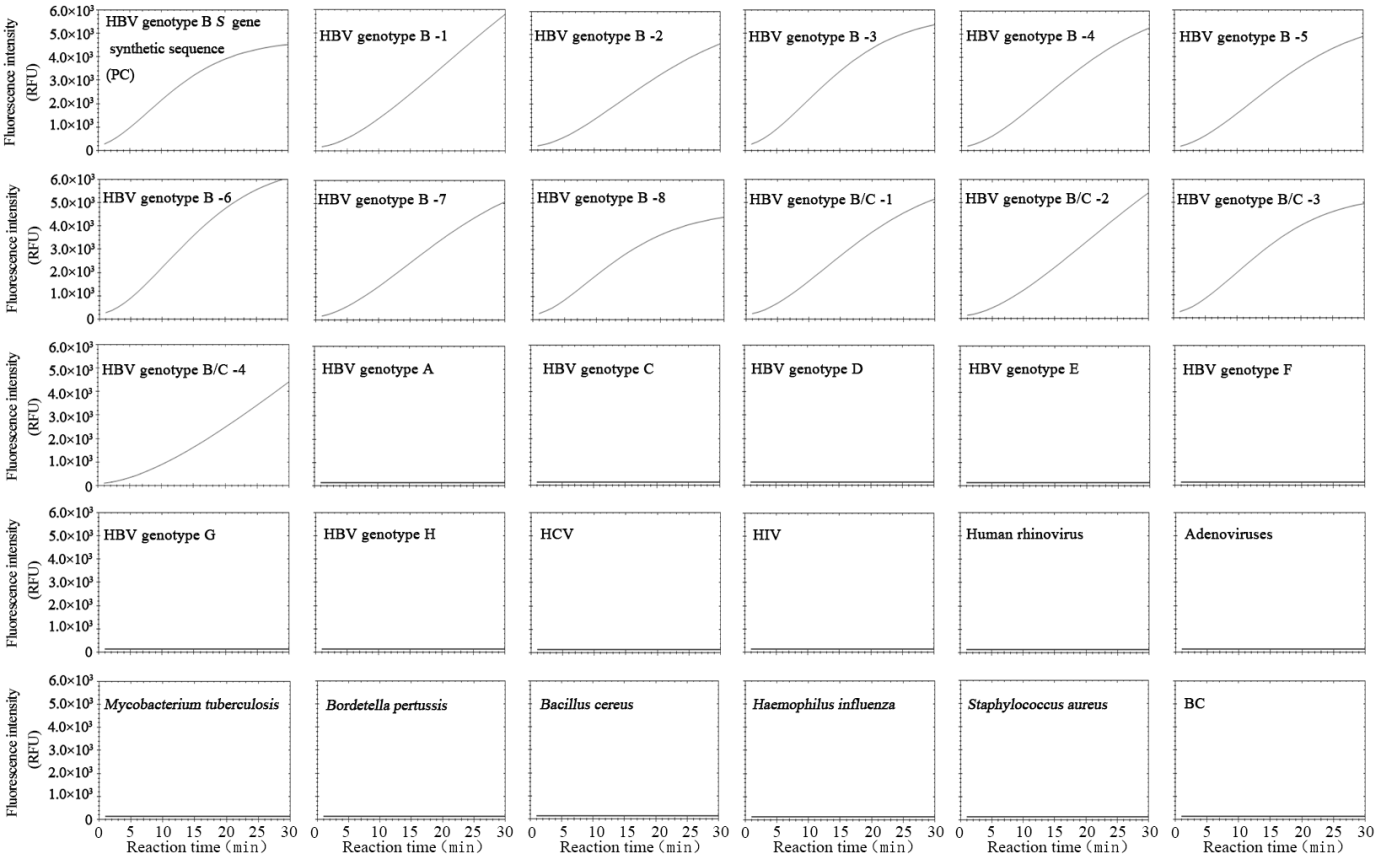


**Fig. S5. The specificity of CRISPR-HBV real-time fluorescence assay for HBV genotype B detection**. The MCDA amplification and CRISPR-Cas12b-based real-time fluorescence detection as described above. The synthesis sequence of HBV genotype B *S* gene has been used as positive control (PC). HBV genotype B or B/C positive patient samples resulted in robust fluorescence curves indicating presence of the HBV genotype B *S* gene. No signal was detected in the other HBV genotypes (A, C, D, E, F, G, and H) and pathogens. PC, positive control; BC, blank control.


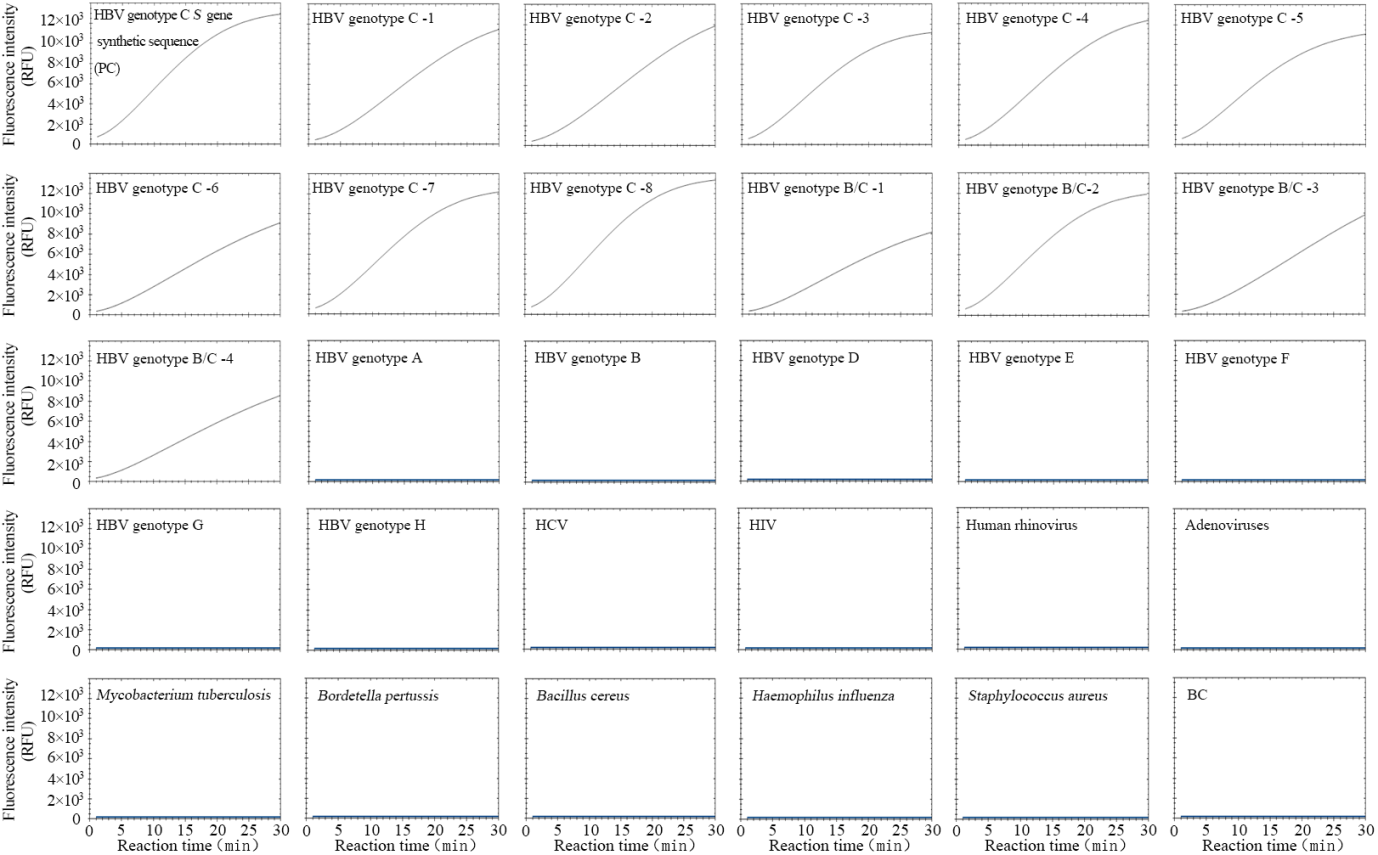


**Fig. S6. The specificity of CRISPR-HBV real-time fluorescence assay for HBV genotype C detection**. The MCDA amplification and CRISPR-Cas12b-based real-time fluorescence detection as described above. The synthesis sequence of HBV genotype C *S* gene has been used as positive control (PC). HBV genotype C or B/C positive patient samples resulted in robust fluorescence curves indicating presence of the HBV genotype B *S* gene. No signal was detected in the other HBV genotypes (A, B, D, E, F, G, and H) and pathogens. PC, positive control; BC, blank control.
